# COVID-19 vaccine hesitancy among healthcare workers in Arab Countries: A systematic review and meta-analysis

**DOI:** 10.1371/journal.pone.0296432

**Published:** 2024-01-02

**Authors:** Mai Alalawi, Muath A. Alsalloum, Yusuf M. Garwan, Mya Abuzeid, Hassan Alalawi, Khalid Eljaaly, Abrar K. Thabit, Jimmy Jose

**Affiliations:** 1 Pharmaceutical Care Department, King Abdulaziz Medical City, Jeddah, Saudi Arabia; 2 Department of Pharmaceutical Sciences, Fakeeh College for Medical Sciences, Jeddah, Saudi Arabia; 3 Department of Pharmacy Practice, College of Clinical Pharmacy, Imam Abdulrahman Bin Faisal University, Dammam, Saudi Arabia; 4 Pharmacy Practice Department, Faculty of Pharmacy, King Abdulaziz University, Jeddah, Saudi Arabia; 5 Faculty of Medicine, King Abdulaziz University, Jeddah, Saudi Arabia; 6 School of Pharmacy, University of Nizwa, Nizwa, Sultanate of Oman; King Saud University College of Medicine, SAUDI ARABIA

## Abstract

**Background:**

Vaccine hesitancy is a major obstacle to the large efforts made by governments and health organizations toward achieving successful COVID-19 vaccination programs. Healthcare worker’s (HCWs) acceptance or refusal of the vaccine is an influencing factor to the attitudes of their patients and general population. This study aimed to report the acceptance rates for COVID-19 vaccines among HCWs in Arab countries and identify key factors driving the attitudes of HCWs in the Arab world toward vaccines.

**Methods:**

This systematic review and meta-analysis followed the PRISMA guidelines. PubMed and Scopus databases were searched using pre-specified keywords. All cross-sectional studies that assessed COVID-19 vaccine hesitancy and/or acceptance among HCWs in Arab countries until July 2022, were included. The quality of the included studies and the risk of bias was assessed using the JBI critical appraisal tool. The pooled acceptance rate of the COVID-19 vaccine was assessed using a random-effects model with a 95% confidence interval.

**Results:**

A total of 861 articles were identified, of which, 43 were included in the study. All the studies were cross-sectional and survey-based. The total sample size was 57,250 HCWs and the acceptance rate of the COVID-19 vaccine was 60.4% (95% CI, 53.8% to 66.6%; *I*^*2*^, 41.9%). In addition, the COVID-19 vaccine acceptance rate among males was 65.4% (95% CI, 55.9% to 73.9%; *I*^*2*^, 0%) while among females was 48.2% (95% CI, 37.8% to 58.6%; *I*^*2*^, 0%). The most frequently reported factors associated with COVID-19 vaccine acceptance were being male, higher risk perception of contracting COVID-19, positive attitude toward the influenza vaccine, and higher educational level. Predictors of vaccine hesitancy most frequently included concerns about COVID-19 vaccine safety, living in rural areas, low monthly income, and fewer years of practice experience.

**Conclusion:**

A moderate acceptance rate of COVID-19 vaccines was reported among HCWs in the Arab World. Considering potential future pandemics, regulatory bodies should raise awareness regarding vaccine safety and efficacy and tailor their efforts to target HCWs who would consequently influence the public with their attitude towards vaccines.

## Introduction

The coronavirus disease of 2019 (COVID-19) pandemic has resulted in a huge negative impact on global health and the economy. Even though hygienic and behavioral control measures are effective in tackling pandemics, vaccines have been proposed as the single most important method to provide protection and control the spread of the virus [1]. The World Health Organization (WHO) estimates that vaccinations prevent between 3.5 and 5 million deaths every year [2]. Since the start of the pandemic and the spread of the virus, efforts to create vaccines have been accelerated to prevent and control the disease.

Vaccine hesitancy has been considered by the WHO as a global health threat in 2019 as it is deemed a barrier to the success of vaccination programs [3]. Vaccine hesitancy has been defined by the Strategic Advisory Group of Experts on Immunization (SAGE) as “a delay in acceptance or refusal of vaccination despite the availability of vaccination services” [4]. There are multiple determinants that influence the attitude in relation to the acceptance of vaccination, including complacency, convenience, and confidence [4]. Multiple previous studies indicated that vaccine acceptance, in general, is declining globally, even though it has been proven that vaccines are safe and effective in controlling the spread and reducing mortality rates [5–9]. The three most common reasons for vaccine hesitancy are risk vs. benefit concerns, lack of awareness and knowledge of vaccinations and their importance, and certain religious and cultural beliefs [7].

In terms of COVID-19, surveys conducted prior to the availability of COVID-19 vaccines in the United States showed that 70% of residents planned to receive the vaccine when available [10]. On the other hand, an international study that investigated the attitudes of healthcare workers (HCWs) toward COVID-19 vaccination found that the rates of high acceptance, moderate acceptance, and hesitancy to receive the vaccine were 48.6%, 23%, and 28.4%, respectively. In addition, 40.88% agreed that the concern about vaccine safety was the most prevalent factor for vaccine hesitancy [11].

Recent studies have demonstrated multiple predictors of COVID-19 vaccine acceptance and/or hesitancy among HCWs. In a systematic review and meta-analysis, Ackah et. al. estimated Covid-19 vaccine acceptance in Africa among HCWs and the associated hesitancy predictors. The study reported a low acceptance rate of 46% that was associated with concerns about vaccines’ safety and efficacy, limited data, and expedited COVID-19 vaccine clinical trials [12].

Vaccine hesitancy and low acceptance rates are major obstacles to the large efforts made by governments and health organizations toward achieving successful COVID-19 vaccination programs [13, 14]. It is important that HCWs receive the vaccine and promote receiving it as they are on the frontlines in defending against COVID-19, which puts them at risk of being disease victims, as well as disease transmitters. This risk arises from frequent contact with COVID-19 patients, visitors, and other HCWs [15]. Moreover, HCWs are considered a reliable and trustworthy source of information by their patients and the public, which makes their acceptance or refusal of the vaccine an influencing factor for the attitudes of their patients and the general population toward receiving the vaccine [16]. Several studies from Arab World countries surveyed HCWs regarding their perception and attitude toward receiving the COVID-19 vaccine. This systematic review and meta-analysis aimed to report the acceptance rates for COVID-19 vaccines among HCWs in Arab countries and identify the predictors of vaccine acceptance. Findings from this review should benefit governments, decision-makers, and other HCWs in identifying key factors driving the attitudes of HCWs in the Arab world toward vaccines and inform decisions that can help change vaccine hesitancy to acceptance of booster doses for COVID-19, future pandemics, as well as when new critical vaccines are introduced into the market.

## Methods

The reporting of this systematic review and meta-analysis was done in line with the Preferred Reporting Items for Systematic Reviews and Meta-Analysis (PRISMA) guidelines [17]. The methodology was based on the manual for evidence synthesis by the Joanna Briggs Institute (JBI) [18].

### Search strategy and study selection

PubMed and Scopus databases were searched independently by MA and AKT using the following keywords: (COVID-19 OR SARS-CoV-2 OR coronavirus) AND (vaccin* OR immunization* OR immunisation*) AND (hesitan* OR reluct* OR refus* OR accept* OR willing* OR intent* OR intend* OR reject* OR delay*) AND (Algeria OR Bahrain OR Comoros OR Djibouti OR Egypt OR Iraq OR Jordan OR Kuwait OR Lebanon OR Libya OR Mauritania OR Morocco OR Oman OR Palestine OR Qatar OR Saudi OR Somalia OR Sudan OR Syria OR Tunisia OR Emirates OR Yemen OR Arab*). The title and abstract screening process was performed by MA and AKT. Disagreements were handled via a discussion with the other study authors. All quantitative cross-sectional studies that assessed COVID-19 vaccine hesitancy and/or acceptance among HCWs in Arab countries up till July 25, 2022, were included. Qualitative (interview-based) studies and studies that included both HCWs along with the general public or students at schools of health sciences without reporting subgroup findings of HCWs were excluded.

### Data extraction

Using a standardized data extraction form, data extraction was executed independently by five authors, MA, MAA, YG, HA, and MJA. The extracted data included the primary author’s name, year of publication, country, the profession of study participants, number of participants accepting the vaccine, the total number of participants, and predictors of vaccine hesitancy and acceptance. Controversies were resolved after a discussion with AKT, JJ, and KE. The data extraction process was revised independently by AKT and JJ.

### Quality assessment

The quality of the included studies and the risk of bias was assessed using the JBI critical appraisal tool (18) by the same authors who performed the data extraction. Disagreements were handled via a discussion with AKT, KE, and JJ. The quality assessment process was revised by AKT.

### Meta-analysis and data synthesis

The pooled acceptance rate of COVID-19 vaccine was assessed using a random-effects model with a 95% confidence interval (95% CI). Heterogeneity (I^*2*^) was assessed using Cochrane’s Q test. Subgroup analyses were performed to report the acceptance rate per country.

## Results

### Search results

**Fig 1:**
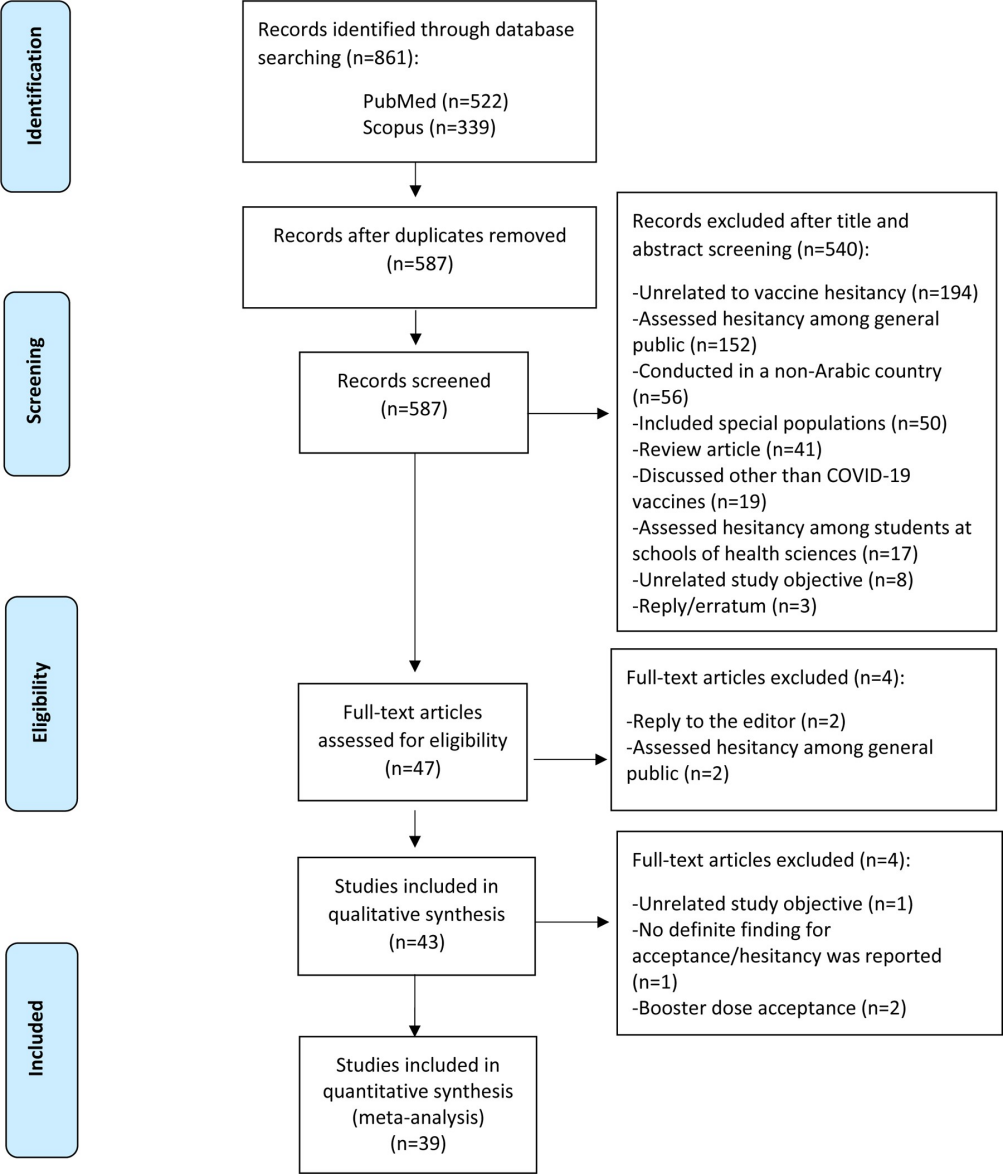
PRISMA flowchart of literature search and review.

A total of 861 records were identified, where only 47 full-text articles met the inclusion criteria. Of the 47 full-text articles screened, 43 and 39 studies were included in the qualitative and quantitative synthesis, respectively. The reasons for excluding the other 514 studies are explained in the PRISMA flowchart (Fig 1). Four of the 47 eligible studies were excluded from the meta-analysis due to the following reasons: assessed hesitancy toward the booster dose exclusively [19, 20] reported vaccine hesitancy to each available vaccine rather than to the concept of vaccination as a whole [21], and assessed hesitancy using several questions with varied answers to each question [22].

### Characteristics of the included studies

The included studies are summarized in Table 1. All 39 included quantitative studies were cross-sectional and survey-based and were distributed to HCWs between July 2020 and November 2021. Most of the data were collected after December 2020 when the vaccines were available in many countries.

**Table 1:** The included studies for the quantitative analysis.

	Author	Country	Date of the survey	Profession of study participants	Number of Participants Accepting the Vaccine	Total Number of Participants (i.e., Sample Size)
1	Alhofaian et al.	SA	Mar 2021 –Apr 2021	Non-specific	298	383
2	Arif et al.	SA	Jul 2021 –Sep 2021	Non-specific	529	529
3	Baghdadi et al.	SA	Jul 2020 –Sep 2020	Non-specific	222	363
4	Barry et al.	SA	Nov 2020	Non-specific	1058	1512
5	Elharake et al.	SA	Oct 2020 –Dec 2020	Non-specific	15299	23582
6	Hershan et al.	SA	Dec 2020	Non-specific	104	186
7	Maqsood et al.	SA	April 2021 –May 2021	Non-specific	996	1031
8	Qattan et al.	SA	Dec 2020	Non-specific	340	673
9	Temsah et al.	SA	Dec 2020 –Jan 2021	Non-specific	352	1058
10	Yassin et al.	Sudan	April 2021	Non-specific	254	400
11	Al Awaidy et al.	Oman	Dec 2020	Non-specific	264	608
12	Khamis et al.	Oman	Jan-Feb 2021	Non-specific	176	433
13	Maraqa et al.	Palestine	Dec 2020-Jan 2021	Non-specific	438	1159
14	Belkebir et al.	Palestine	Jan 2021	Nurses	15	46
15	Rabi et al.	Palestine	Jan 2021	Nurses	517	638
16	Kumar et al.	Qatar	Oct-Nov 2020	Non-specific	619	1414
17	Zammit et al.	Tunisia	Jan 2021	Non-specific	237	493
18	Albahri et al.	UAE	Jul 2020	Physicians and nurses	104	176
19	AlKetbi et al.	UAE	Nov 2020-Feb 2021	Non-specific	2525	2832
20	Saddik et al.	UAE	Nov 2020 ‐ Jan 2021	Non-specific	312	517
21	Aloweidi et al.	Jordan	Jan 2021 ‐ Feb 2021	Non-specific	131	287
22	Lataifeh et al.	Jordan	Feb2021- March 2021	Physicians and Nurses (including midwives)	233	364
23	Qunaibi et al.	Multi-national	14–29 Jan 2021	Non-specific	1522	5708
24	Al-Sanafi et al.	Kuwait	18–29 March 2021	Non-specific	849	1019
25	Nasr et al.	Lebanon	15–22 Feb 2021	Dentists	455	529
26	Youssef et al.	Lebanon	10–31 Dec 2020	Non-specific	1044	1800
27	Elhadi et al.	Libya	1–18 Dec 2020	Non-specific	1781	2215
28	Khalis et al.	Morocco	First 3 weeks of Jan 2021	Non-specific	119	170
29	Ahmed et al.	SA	Not mentioned	Non-specific	131	236
30	Aldosary et al.	SA	Nov 2020 ‐ Dec 2020	Nurses	236	334
31	Hammam et al.	Egypt	1 week during April 2021	Rheumatologist	57	187
32	Fares et al.	Egypt	Dec 2020 ‐ Jan 2021	Non-specific	81	385
33	Elkhayat et al.	Egypt	Jun 2021 ‐ Aug 2021	Non-specific	142	341
34	El-Sokkary et al.	Egypt	January 25 ‐ 31, 2021	Non-specific	80	308
35	El Kibbi et al.	Arab World	April 13 ‐ May 11, 2021	Non-specific	1237	1517
36	Noushad et al.	SA	Feb 2021 ‐ March 2021	Non-specific	433	674
37	Shehata et al.	Egypt	March 2021 –May 2021	Physicians	495	1268
38	Sharaf et al.	Egypt	Aug 2021 –Oct 2021	dental faculty	78	171
39	Luma et al.	Iraq	Feb 2021	Non-specific	1229	1704

SA, Saudi Arabia; UAE, United Arab Emirates, Non-specific, included more than two healthcare professions.

Most of the studies were from Saudi Arabia (n = 15) [19–21, 23–34] followed by Egypt (n = 6) [35–40], Jordan (n = 3) [22, 41, 42], Palestine (n = 3) [43–45], United Arab Emirates (n = 3) [46–48], Iraq (n = 2) [49, 50], Lebanon (n = 2) [51, 52], and Oman (n = 2) [53, 54]. Only one study was found for each of the following countries: Kuwait [55], Libya [56], Morocco [57], Qatar [58], Sudan [59], and Tunisia [60]. No studies were found from the remaining eight Arab countries (Algeria, Bahrain, Yemen, Somalia, Syria, Mauritania, Djibouti, and Comoros Islands). Two studies reported vaccine hesitancy in HCWs among different Arab countries. Firstly, the ARCOVAX study which is a large cross-sectional study conducted among HCWs in 19 Arab countries [61]. Another study by Qunaibi et al. included Arabic-speaking HCWs residing inside and outside Arab countries [62].

The sample size per study ranged from 46 to 23,582 (median = 568) and the total sample size was 57,250. Of the chosen studies, 27 included < 1000 participants, 15 included 1000–3000 participants, and two included > 3000 participants.

While most studies targeted more than two HCWs professions (n = 35), others targeted the following: physicians and nurses (n = 2) [42, 46]; dentists/dental faculty (n = 2) [39, 51]; nurses (n = 3) [33, 44, 45]; rheumatologists (n = 1) [35]; and physicians (n = 1) [40].

### The methodological quality of the included studies

S1 Table represents the quality assessment of the included studies using the JBI tool. The risk of bias was low for 37 studies and medium for seven studies [20, 23, 33, 35, 38, 40, 44].

### COVID-19 vaccine acceptance rate in Arab countries

**Fig 2:**
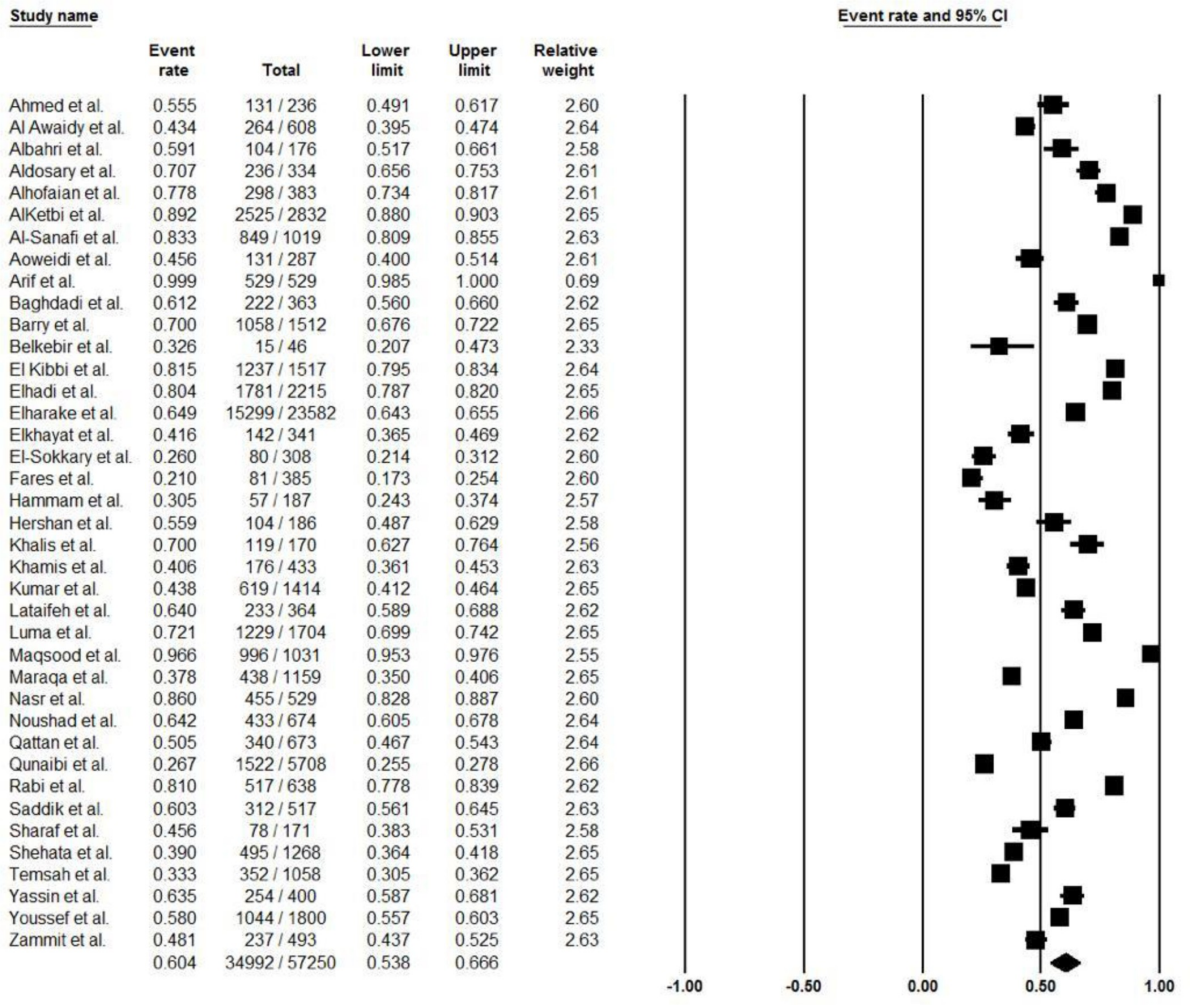
The overall acceptance rate of COVID-19 vaccines among Arab countries.

**Fig 3:**
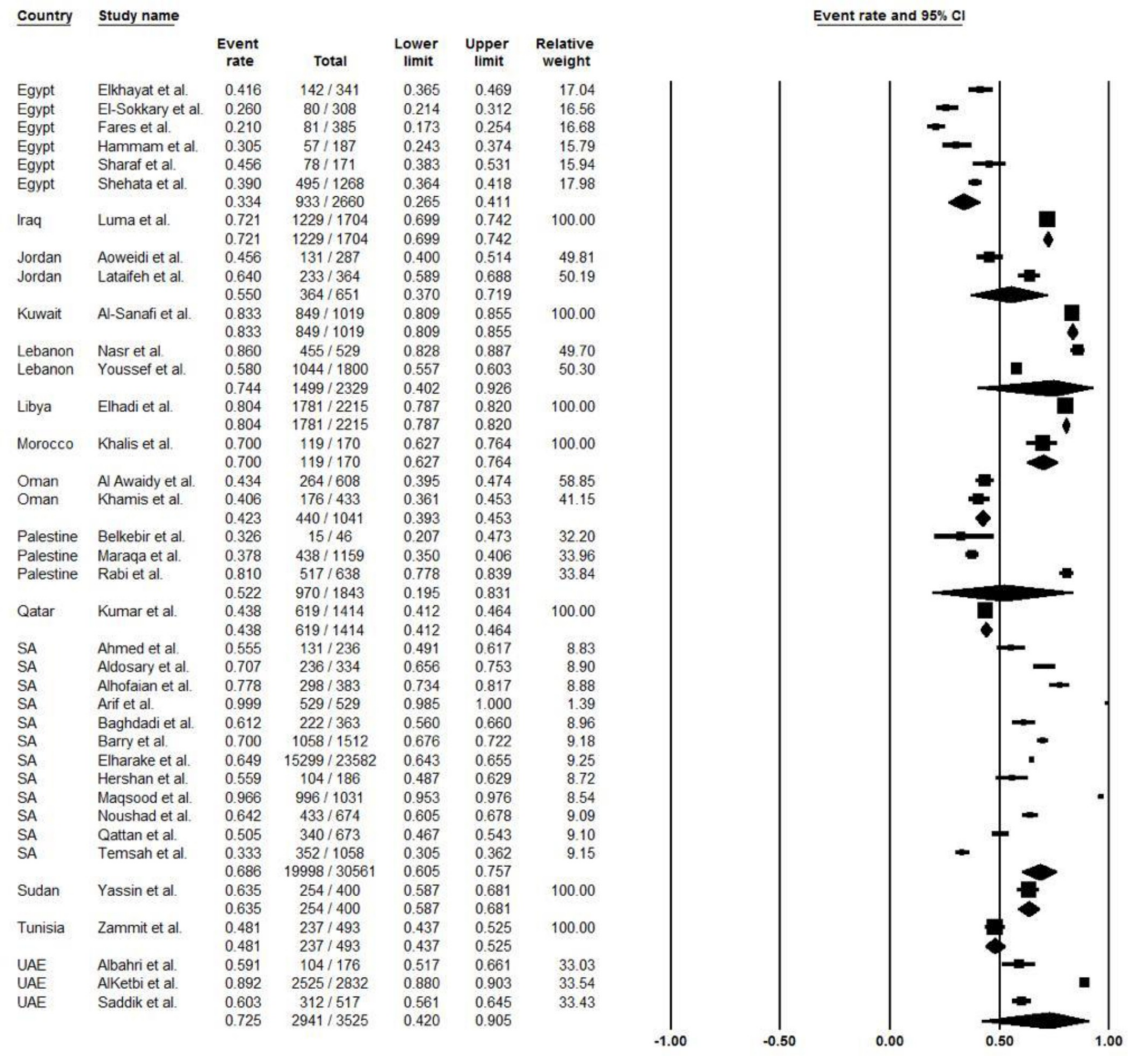
The acceptance rate of COVID-19 vaccines by country.

**Fig 4:**
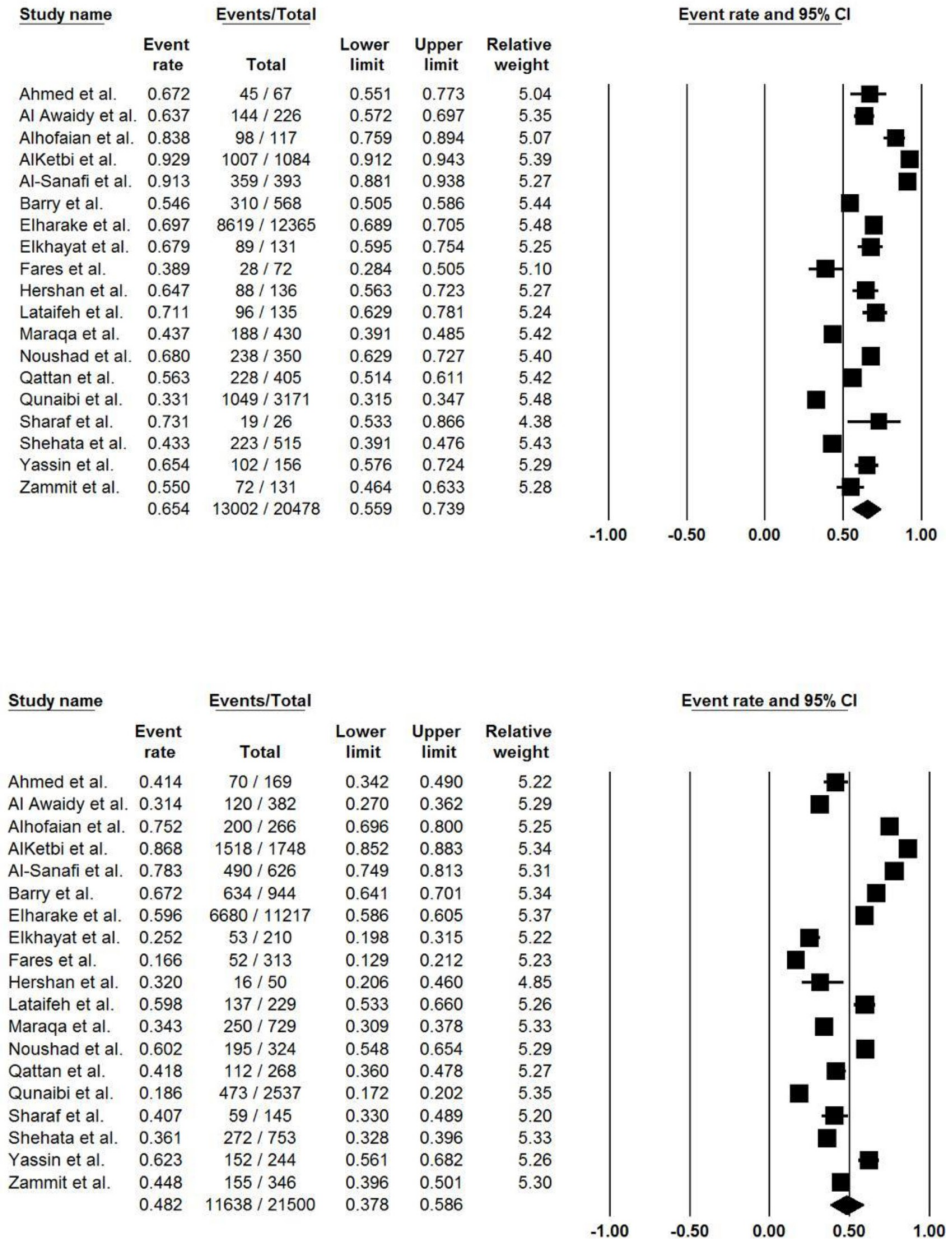
COVID-19 vaccine acceptance stratified by sex. (A) males (B) females.

The pooled acceptance rate of COVID-19 vaccine was 60.4% (95% CI, 53.8% to 66.6%; *I*^*2*^, 41.9%) (Fig 2). The pooled vaccine acceptance rate estimated for each country was as follows: Egypt 33.4% (95% CI, 26.5% to 41.1%; *I*^*2*^, 13.7%), Jordan 55.0% (95% CI, 37.0% to 71.9%; *I*^*2*^, 0%), Lebanon 74.4% (95% CI, 40.2% to 92.6%; *I*^*2*^, 0%), Oman 42.3% (95% CI, 39.3% to 45.3%; *I*^*2*^, 0%), Palestine 52.2% (95% CI, 19.5% to 83.1%; *I*^*2*^, 0%), Saudi Arabia 68.6% (95% CI, 60.5% to 75.7%; *I*^*2*^, 74.9%), and United Arab Emirates 72.5% (95% CI, 42.0% to 90.5%; *I*^*2*^, 0%) (Fig 3). In addition, the COVID-19 vaccine acceptance rate among males was 65.4% (95% CI, 55.9% to 73.9%; *I*^*2*^, 0%) while among females was 48.2% (95% CI, 37.8% to 58.6%; *I*^*2*^, 0%) (Table 2), (Fig 4).

**Table 2:** COVID-19 vaccine acceptance among HCWs stratified by sex.

	Author	Country	Number of Participants Accepting the Vaccine			Total Number of Participants (i.e., Sample Size)		
			Total	Male	Female	Total	Male	Female
1.	Ahmed et al.	SA	131	45	70	236	67	169
2.	Al Awaidy et al.	Oman	264	144	120	608	226	382
3.	Albahri et al.	UAE	104	**NR**	**NR**	176	18	158
4.	Aldosary et al.	SA	236	**NR**	**NR**	334	25	309
5.	Alhofaian et al.	SA	298	98	200	383	117	266
6.	AlKetbi et al.	UAE	2525	1007	1518	2832	1084	1748
7.	Al-Sanafi et al.	Kuwait	849	359	490	1019	393	626
8.	Aloweidi et al.	Jordan	131	**NR**	**NR**	287	97	190
9.	Arif et al.	SA	529	**NR**	**NR**	529	167	362
10.	Baghdadi et al.	SA	222	107	115	363	**NR**	**NR**
11.	Barry et al.	SA	1058	310	634	1512	568	944
12.	Belkebir et al.	Palestine	15	**NR**	**NR**	46	13	33
13.	EI Kibbi et al.	Arab World	1237	**NR**	**NR**	1517	**NR**	**NR**
14.	Elhadi et al.	Libya	1781	**NR**	**NR**	2215	743	1472
15.	Elharake et al.	SA	15299	8619	6680	23582	12365	11217
16.	Elkhayat et al.	Egypt	142	89	53	341	131	210
17.	EI-Sokkary et al.	Egypt	80	29	51	308	**NR**	**NR**
18.	Fares et al.	Egypt	81	28	52	385	72	313
19.	Hammam et al.	Egypt	57	**NR**	**NR**	187	28	159
20.	Hershan et al.	SA	104	88	16	186	136	50
21.	Khalis et al.	Morocco	119	**NR**	**NR**	170	**NR**	**NR**
22.	Khamis et al.	Khamis	176	**NR**	**NR**	433	139	304
23.	Kumar et al.	Qatar	619	**NR**	**NR**	1414	**NR**	**NR**
24.	Lataifeh et al.^$^	Jordan	233	96	137	364	135	229
25.	Luma et al.	Iraq	1229	**NR**	**NR**	1704	978	726
26.	Maqsood et al.	SA	996	**NR**	**NR**	1031	281	750
27.	Maraqa et al.	Palestine	438	188	250	1159	430	729
28.	Nasr et al.	Lebanon	455	**NR**	**NR**	529	292	237
29.	Noushad et al.	SA	433	238	195	674	350	324
30.	Qattan et al.	SA	340	228	112	673	405	268
31.	Qunaibi et al.	multinational	1522	1049	473	5708	3171	2537
32.	Rabi et al.	Palestine	517	**NR**	**NR**	638	115	523
33.	Saddik et al.	UAE	312	**NR**	**NR**	517	187	330
34.	Sharaf et al.	Egypt	78	19	59	171	26	145
35.	Shehata	Egypt	495	223	272	1268	515	753
36.	Temsah et al.	SA	352	**NR**	**NR**	1058	354	704
37.	Yassin et al.	Sudan	254	102	152	400	156	244
38.	Youssef et al.	Lebanon	1044	**NR**	**NR**	1800	593	1209
39.	Zammit et al.	Tunisia	237	72	155	493	131	346

NR = Not reported and was excluded from the quantitative analysis; SA, Saudi Arabia; UAE, United Arab Emirates; $ not statistically significant.

### Vaccine acceptance by country

Table 3 summarizes the factors associated with vaccine acceptance. Only factors that showed a significant association with acceptance (*P* < 0.05) among HCWs were listed. A total of 33 studies identified various predictors for COVID-19 vaccine acceptance. The most commonly reported predictor was sex (n = 24), where in 91.6% of the studies, male healthcare workers were more likely to accept the vaccine than female. Other commonly reported predictors were a high perception of getting COVID-19 (n = 14), belief in the COVID-19 vaccine’s benefit (n = 11), positive attitude towards influenza vaccine (n = 11), high level of knowledge regarding COVID-19 disease/vaccine (n = 9), use of a reliable source of information (n = 5), the belief that the vaccine should be mandatory (n = 4), and work in frontline position (n = 4).

**Table 3:** Significant predictors of COVID-19 vaccine acceptance among HCWs reported in each study.

	Predictors of Vaccine Acceptance										
No.	Author/year, country	Reference No.	Variables (Statistically Significant)								
			Age group	Sex	Marital Status	Education level	Presence of comorbidities	Flu vaccine	COVID risk/ susceptibility perception	Vaccine perceived benefit	Others
1.	Alobaidi/2022, SA*			Male	Widow/divorced/separated	Higher (MS, PhD)	✔				• Nationality: Non-Saudi • Monthly income: higher (>15K SR)
2.	Arif/2021, SA			Female	Married	Bachelor					• Monthly income: moderate (5K-10K SR) • Vaccine convenience (vaccination method, frequency, distance to vaccination site. etc) • Higher number of vaccinated friends/family members • Belief that vaccine should be mandated by the government
3.	Baghdadi/2021, SA		Middle (30–49 yr)	Female					✔	✔	• Perceived severity of COVID-19 • Belief that all HCWs should be vaccinated against COVID-19 • Work experience: low (<5 yr) • Non-smokers • Having no fear of injections • Perceived lack of barriers to receive the vaccine
4.	Barry/2021, SA			Male	Married			✔	✔	✔	• Working in adult ICU and isolated floors • Source of information: CDC
5.	Elharake/2021, SA		Younger (25–34 yr)	Male							• Nationalities: Non-Saudi
6.	Maqsood/2022, SA			Male						✔	• Nationalities: Non-Saudi
7.	Qattan/2021, SA			Male				✔	✔		• Being a frontline healthcare worker • Believe that COVID19 vaccine should be compulsory
8.	Al Awaidy/2020, Oman			Male						✔	• Recommend COVID-19 to family members • Higher level of knowledge about COVID-19 vaccine • Trust in government • Positive attitude towards COVID-19 vaccine
9.	Khamis/2022, Oman			Male						✔	Belief that vaccine should be mandated
10.	Maraqa/2021, Palestine		Younger	Male				✔	✔		• Physicians • Working in a non-governmental setting • Higher knowledge about COVID-19 • - High income
11.	Rabi/2021, Palestine		Younger								• Higher level of knowledge about COVID-19 vaccine • Perception of the vaccine as safe • Preference of vaccination over natural immunity • No fear of injections • Not influenced by media misrepresentation • COVID-19 might cause or potentiate existing chronic diseases
12.	Kumar/2021, Qatar		26–35 yr	Male	Married			✔	✔	✔	• Higher level of knowledge about COVID-19 and the vaccine • Recommending the vaccine to oneself children
13.	Zammit/2022, Tunisia		≥ 40 yr	Male							• Works in North of Tunisia • Previously infected with COVID-19 • The national and official health websites as the primary source for COVID-19-related information
14.	Albahri/2021, UAE										• Nationality: non-Emirati • Profession: Nurses
15.	AlKetbi/2021, UAE		Older	Male							• Physicians/surgeons • Working in private sector • Nationality: South Asian • Religion
16.	Saddik/2022, UAE			Male				✔	✔		• Positive attitude towardCOVID-19 vaccine • Belief that there are sufficient data about the vaccine • No concern about vaccine side effects
17.	Aloweidi /2021, Jordan				Unmarried				✔	✔	• Medical personnel advice • Social media awareness campaigns • Reading a scientific article about the available vaccines • Trust in vaccine efficacy and safety • National medical studies to prove the efficacy of the vaccines
18.	Lataifeh/2022, Jordan							✔			• Being a physician (vs. nurses) • Registering on COVID-19 vaccine special platform
19.	Qunaibi/2021, multinational		Older	Male				✔			
20.	Al-Sanafi/2021, Kuwait			Male		Postgraduate degree					• Being a physician or dentist • Nationality: Kuwaiti • Working in public sector
21.	Nasr/2021, Lebanon							✔	✔		• Moderate or high knowledge level regarding COVID-19 vaccine • fear of COVID-19 disease
22.	Youssef/2022, Lebanon			Male				✔		✔	• Frontline health workers • Concerns about limited accessibility to the vaccine • Concerns about availability of the vaccine. • Receiving reliable and adequate information about vaccine • Recommendation from health authorities and health facilities
23.	Khalis/2021, Morocco										• Being a physician, nurse, or technician • high score of confidence in the information circulating about COVID-19 • high score of perceived severity of COVID-19
24.	Ahmed/2021, Saudi Arabia		• 36 > years	Female			✔				
25.	Aldosary/2021, Saudi Arabia										• Higher knowledge score about COVID-19 disease • Higher practice score toward COVID-19 measures
26.	Alhasan/2021, Saudi Arabia*										• Saudi National • Receiving both vaccination doses (1^st^ and 2^nd^ shots) • High knowledge score about delta variant. • High perception score regarding COVID-19 measures (mask, lockdown..) • Believing that the Pfizer-BioNTech BNT162b2 vaccine is effective against Delta variant. • Agreement that mixing/matching vaccines is effective against Delta variant.
27.	Fares/2021, Egypt			Male					✔		Those who took non-compulsory vaccines Those who recommended COVID-19 vaccination to others Those who received advice from their hospitals to get the vaccine Those who showed trust in vaccine producers Those who received sufficient information and trusting the information on COVID-19 vaccine Considering taking vaccine as a community responsibility
28.	Elkhayat/2021, Egypt		Younger people (18–<30)	Male	Single	Higher		✔	✔	✔	Using reliable source of information Urban residence Job category- Doctors Attending awareness sections
29.	El-Sokkary/2021, Egypt		42.5 ± 12.2	Male		Higher			✔	✔	• Monthly income: > 5000 LE • > 10 years of working experience • Using reliable sources of information for vaccines and attending vaccine meetings.
30.	Shehata/2022, Egypt		Youngest group (18–30)	Male	Married	Lower (bachelor’s grade)		✔	✔		• Urban area • Physicians who worked in frontline positions • primary surgical and surgical subspecialty • physicians who had someone they knew diagnosed with COVID-19 and had direct contact with COVID-19 patients • Absence of comorbidities • Obesity • COPD • Non-smoking
31.	Sharaf/2022, Egypt			Male					✔	✔	• Not anxious about Covid-19 at all • never postponed other recommended vaccines • has intentions to travel internationally • responsibility and fear of transmitting the disease to relatives or friends • not concerned about the safety and efficacy of vaccines
32.	Noushad/2021, SA		Oldest group (50 and above)	Male					✔		• updating self on the development of COVID-19 vaccines • opinion about the severity of COVID-19 (increased severity perception) • Greater compliance with COVID-19 preventive guidelines • higher level of anxiety about contracting COVID-19 • Saudi nationals
33.	Hamdan- Mansour /2022, Jordan			Male							• History of COVID-19 infection • Higher level of knowledge and perception about COVID-19 vaccines.

*Evaluated the acceptance of the COVDI-19 vaccine booster dose.

#### Saudi Arabia

Vaccine acceptance among HCWs in Saudi Arabia was assessed in 15 national [19–21, 23–34], and two multinational [61, 62] studies. All the studies were conducted during the period between July 2020 and November 2021, and seven studies were conducted before the introduction of COVID-19 vaccines in Saudi Arabia (i.e., before December 2020) [21, 25–28, 30, 33].

Certain sociodemographic factors were linked to vaccine acceptance, including being male (n = 7) [26, 27, 29, 30, 32, 34, 62], being older (n = 2) [30, 62], and being married (n = 2) [24, 26]. Other predictors of vaccine acceptance included a high-risk perception of getting COVID-19 (n = 4) [25, 26, 30, 34], belief in the COVID-19 vaccine’s benefit (n = 3) [25, 26, 29], positive attitudes towards influenza vaccine (n = 3) [26, 30, 62], belief that the vaccine should be mandatory (n = 3) [24, 25, 30], vaccine convenience/lack of barriers towards receiving the vaccines, (n = 2) [24, 25], working directly in high-risk settings (n = 2) [26, 30], and history of chronic illness (n = 1) [32].

Two studies evaluated the prevalence and predictors of booster dose acceptance among HCWs in Saudi Arabia and reported vaccine acceptance rates between 55–71% [19, 20]. Alhasan et al. identified certain factors associated with higher acceptance for booster doses, including complete primary COVID-19 vaccination (1st and 2nd doses), knowledge about the delta variant, and belief in vaccines’ effectiveness against the delta variant [20].

#### Egypt

Six national studies [35–40] and one multinational study [61] evaluated COVID-19 vaccine acceptance among HCWs in Egypt. The studies took place between December 2020 and October 2021. This period covers the phase before and after the availability of vaccines in the country [35–40]. All the studies surveyed several HCWs of different professions; however, only a few included specific professions, such as dental faculty members [39] and rheumatologists [35].

Several sociodemographic factors were associated with vaccine acceptance. The most reported were male sex (n = 5) [36–40], younger age groups of 18–30 years (n = 2) [37, 40], and higher educational level (n = 2) [37, 38]. In addition, other factors were correlated with vaccine acceptance, including COVID-19 risk susceptibility perception (n = 5) [36–40], previous receipt of flu vaccine (n = 2) [37, 40], and perceived vaccine benefit (n = 2) [37, 38].

Among physicians, Shehata et. al. described more factors that were significantly associated with their vaccine acceptance, particularly physicians working in frontline positions, physicians with surgical specialty, personally knowing someone diagnosed with COVID-19, and absence of medical comorbidity [40].

#### Jordan

In addition to the multinational study by El Kibbi, et al [61], a total of three studies were conducted in Jordan. Of these, two were conducted between January 2021 to March 2021 [41, 42] while one did not specify the study period [22]. Aloweidi et al. compared between HCWs (defined as medical personnel working in direct contact with patients) vs non-medical personnel in terms of the factors that could predict vaccine acceptance. The factors that significantly influenced HCWs to receive the vaccines included awareness campaigns on social media, advice from other HCWs, availability of national studies proving the efficacy of the vaccine, and trust in vaccine efficacy and safety. Moreover, HCWs who read scientific articles reported higher vaccine acceptance rate [41].

#### Palestine

Three studies assessed vaccine hesitancy among HCWs in Palestine [43–45]. Surveys were distributed between December 2020 and January 2021. Two studies were conducted exclusively among nurses [44, 45]. The most frequently reported factors associated with vaccine acceptance were being younger (n = 2) [43, 45] and having higher knowledge about COVID-19 disease/vaccines (n = 2) [43, 45].

#### United Arab Emirates (UAE)

Three studies surveyed HCWs’ COVID-19 acceptance in the UAE from July 2020 to January 2021 [46–48]. In two studies, the male sex was reported as a predictor for vaccine acceptance [47, 48] Furthermore, Saddik et al. reported several other factors including, previously receiving the flu vaccine, COVID-19 risk susceptibility perception, positive attitude toward the vaccine, availability of sufficient data, and having no concerns regarding the side effects of the vaccine [48]. Interestingly, Albahri et al. reported that non-Emirati residents were more willing to receive the vaccine [46], particularly South Asian residents [47].

#### Iraq

Two studies were published in Iraq concerning HCWs’ acceptance of COVID-19 vaccines [49, 50]. However, the latter was excluded from the meta-analysis because they also included the general population in their analysis [50]. On the other hand, Luma et al. identified various factors associated with vaccine hesitancy including female sex, lower level of education, poor health, and pre-existing chronic diseases [49].

#### Lebanon

Two studies were published from Lebanon assessing HCWs’ acceptance of COVID-19 vaccines [51, 52]. The first by Nasr et al. included dentists only. The factors that were associated with vaccine acceptance were previously receiving the flu vaccine, COVID-19 risk susceptibility perception, and moderate to high knowledge level regarding the COVID-19 vaccine [51]. The second study by Youssef et al. surveyed HCWs of different professions and reported that male sex, previous receipt of the flu vaccine, vaccine perceived benefit, frontline health workers’ concerns about limited access and availability to the vaccine, receiving reliable information regarding the vaccine, and recommendations done by health authorities were all positively associated with COVID-19 vaccine acceptance [52].

#### Oman

Vaccine hesitancy among HCWs was assessed in two studies in Oman [53, 54]. One study was conducted in December 2020, before the vaccine rollout in the country [53]. In both studies, males were more likely to accept vaccines than females [53, 54]. Other factors associated with vaccine acceptance included a higher level of knowledge, a positive attitude toward the vaccine, and trusting the government.

#### Kuwait

Only one national study was reported from Kuwait that assessed COVID-19 vaccine acceptance, which was conducted in March 2021 and included HCWs of different professions. The reported predictors for vaccine acceptance were male sex, higher level of education (i.e., post-graduate degree), Kuwaiti citizens, being a physician or dentist, and working in the public sector [55].

#### Libya

Only one national study conducted in December 2020 by Elhadi et al. evaluated the acceptance of the COVID-19 vaccine in both HCWs and the general population in Libya. The authors reported that the odds of accepting the vaccine among HCWs were associated with the age group 31–50 years old and having a friend or relative infected with COVID-19 [56].

#### Morocco

Khalis et al. estimated the vaccine acceptance in Morocco in January 2021. They found that vaccine acceptance was more likely among certain professions, including physicians, nurses, and technicians. Moreover, higher confidence in information regarding the COVID-19 vaccine or high perceived severity of the disease were associated with accepting the vaccine [57].

#### Qatar

Only one study from Qatar evaluated the hesitancy toward COVID-19 vaccines among HCWs [58]. The data were collected between October and November 2020, before the vaccine became available in Qatar. Factors associated with vaccine acceptance were being younger (26–35 years), being male, having positive attitudes towards the influenza vaccine, and having higher knowledge about COVID-19 disease. Concerns about vaccine safety and effectiveness were the most common reasons for vaccine hesitancy [58].

#### Sudan

A multicenter hospital-based cross-sectional study was conducted by Yassin et al in April 2021 to evaluate the knowledge, perception, and acceptability of COVID-19 vaccination among HCWs in Sudan [59]. The study reported a statistically significant association between knowledge about vaccination and professions. Among the participants who were aware of COVID-19 vaccinations, 47.7% were nurses and technicians, 35.7% were physicians, and 16.5% were pharmacists. Most participants referred to social media (47.5%) and hospital announcements (45.3%) for vaccine-related information.

#### Tunisia

Zammit et al. reported vaccine acceptance among HCWs in Tunisia. Notably, the study surveyed different hospital staff, which also included administrators and janitors. The study reported several predictors that were positively associated with vaccine acceptance, including. male sex, age ≥ 40 years, positive history of COVID-19 infection, working in north Tunisia, and utilizing official health websites as a primary source of information related to COVID-19 [60].

#### Arab world

The COVID-19 vaccine acceptance was assessed in a multinational study by El Kibbi et al., which included 19 Arab countries. The study reported that the male sex, older age, higher level of education, previously receiving the flu vaccine, and COVID-19 risk susceptibility perception were all significantly associated with vaccine acceptance [61].

## Discussion

Achieving herd immunity using vaccines is an essential strategy to prevent and control the COVID-19 pandemic, where the populations’ acceptance of them is key. The attitude of HCWs toward COVID-19 vaccines represents a triggering factor to the general population’s acceptance of them [16, 63]. In our analysis, we found that the average acceptance rate of COVID-19 vaccine among HCWs in Arab countries was 60.4%. We also noticed various predictors to be associated with vaccine acceptance, namely male sex, higher educational degree, vaccination against flu, COVID-19 risk susceptibility perception, and vaccine perceived benefit.

The rate of COVID-19 vaccine acceptance among HCWs has been found to be variable from region to region. For instance, Ackah et al., who conducted a systematic review and meta-analysis in Africa, reported a lower rate of COVID-19 vaccine acceptance (46%; 95% CI, 37% to 54%) [12]. This could be potentially attributed to the lower availability of and limited accessibility to COVID-19 vaccines in Africa compared to the Arab region [64]. On the other hand, Bianchi and colleagues, who included 14 studies from Italy, reported a COVID-19 vaccine acceptance rate of 87% [65]. This could be explained by Italy’s higher COVID-19-related morbidity and mortality [64]. Similarly, higher rates of COVID-19 vaccine acceptance were reported from meta-analyses that included studies from different parts of the world [66, 67].

More than half of the studies included in our quantitative analysis surveyed participants from the Gulf Cooperation Council (GCC) countries. A review that included 49 studies from the GCC countries that surveyed the general public reported a vaccine acceptance rate similar to the one reported in our study on HCWs (57%) [68]. Most of the GCC countries’ governments have put in notable efforts and taken effective precautionary measures to combat the pandemic, including distributing clear and continually updated COVID-19 vaccine-related resources [69, 70]. The source of COVID-19 vaccine-related information has been reported to influence the attitude toward the COVID-19 vaccine directly [71]. The similar rate of COVID-19 vaccine acceptance between the general public and HCWs, therefore, is possibly attributed to the official governments’ channels being their primary source of COVID-19-related information.

The most frequently reported factors associated with COVID-19 vaccine acceptance in the included studies were being male (n = 21), higher risk perception of contracting COVID-19 (n = 12), positive attitude toward the influenza vaccine (n = 10), and higher educational level or higher knowledge about COVID-19 vaccine (n = 9). Our results are consistent with previous meta-analyses of global studies [66, 67]. Additionally, Fisher and colleagues, who surveyed adults in the U.S., noted that a higher educational level was associated with COVID-19 vaccine acceptance [72]. Individuals with higher educational levels tend to be lifelong learners. Such correlation with vaccine acceptance might be due to their continuous reading of scientific information from reliable sources to keep themselves updated and well-informed. A potential additional explanation behind the higher rate of acceptance among males is that the majority of HCWs in GCC countries are foreigners who may also have dependents. Hence, since practice licensure maintenance and/or residence status renewal in most GCC countries was linked to the receipt of COVID-19 vaccine, these male HCWs had to accept it. This was evident in a cross-sectional study of 870 participants in the Arab world by Kaadan, et al who noticed that a significantly higher proportion of vaccine acceptors were males and those living outside their home countries (i.e., considered foreigners) [73]. Moreover, different Arab countries had different COVID-19 vaccine products (Russian, Chinese, American, or European) based on governmental agreements. As such, the type of the vaccine offered free of charge to the public and the available circulating information about them in the media and those offered by health authorities may have also influenced the decision to receive the vaccine. For example, in a study by Abu-Farha, et al on 2,925 participants from various Arab countries, participants had a higher preference to receive an American vaccine (i.e., Pfizer/BioNTech and Moderna) vs. other kinds of vaccines [74].

Interestingly, the pooled vaccine acceptance rate was lower among female HCWs in comparison to males (48.2% vs 65.4%, respectively). Such observation could be attributed to the females’ negative perception of vaccines’ side effects on fertility and teratogenicity that could carry serious harm [37, 60]. For instance, a cross-sectional study conducted in Saudi Arabia reported a higher hesitancy rate regarding COVID-19 vaccines among pregnant females and females who were planning for pregnancy [75]. In addition, the same results were supported in several studies among pregnant females [76–78]. Nonetheless, such claims lack generalizability since the reasons behind Arab female HCWs’ vaccine refusal were not studied enough, and the pregnancy status was not mentioned in the included studies. Lastly, some studies included more female participants than males [36, 38–40].

Concerns about COVID-19 vaccine safety were among the most commonly reported predictors of vaccine hesitancy [36, 38, 39, 42, 61]. The same concern was also reported globally among the general population and HCWs [64]. The spread of false information through social media is significantly associated with vaccine hesitancy, where some HCWs rely on social media as a major source of information, followed by scientific journals and television [60]. Furthermore, the issue of vaccine safety arises from the lack of long-term safety trials, the expedited approval of several vaccines, and the lack of trust in pharmaceutical companies [39, 61]. Although those factors were valid during the initial rollout of COVID-19 vaccines, trust in safety and effectiveness were built after the vaccines were administered to the masses. For instance, in the studies that evaluated booster doses vaccine acceptance in Saudi Arabia noted that higher knowledge level about COVID-19 variants and receiving the initial doses were positively associated with higher vaccine acceptance rates [19, 20]. Lastly, the geographic distribution of HCWs was also associated with vaccine hesitancy. For instance, HCWs living in rural areas were more likely to refuse the vaccine compared to those living in urban areas [52]. Moreover, HCWs with low monthly income and fewer years of experience were more likely to reject COVID-19 vaccines [38]. Those results are important for health authorities and vaccine campaigns that should tailor their vaccine promotion messages to suit all categories of HCWs.

### Implications and recommendations

Ministries of health and healthcare institutions should conduct regular continuing medical education sessions concerning the development processes of vaccines as well as their safety and effectiveness throughout history. Such sessions should be centered to address the concerns and queries that HCWs might have, including the rationale behind the expedited process of COVID-19 vaccine approval as well as illustrating the precautionary measures taken by the legislative agencies to ensure meeting the standards for safety and efficacy. Regulatory bodies could tailor their efforts to target HCWs who have a negative attitude toward the influenza vaccine or vaccines in general, particularly those with a lower level of postgraduate training or education, given their higher tendency to be vaccine-hesitant. Additionally, HCWs should be encouraged to keep up with scientific publications as this has been found to be associated with higher rates of vaccine acceptance [41].

### Strengths and limitations

This systematic review and meta-analysis is the first that reported the acceptance rate of COVID-19 vaccines by HCWs in the Arab World and examined the potential predictors of such outcome. We critically evaluated the evidence using a valid assessment tool to avoid biased estimates. We also limited the meta-analysis to studies that included only the targeted population. Nonetheless, our study has some limitations. First, all the included studies were self-administered, cross-sectional, survey-based studies, which implies that our findings could have been impacted by a social desirability bias (i.e., the tendency of participants to answer socially acceptable answers rather than those that reflect their true feelings and practices); hence, limits their generalizability. Second, about 35% of the included studies in the meta-analysis surveyed participants from Saudi Arabia, and no studies were reported from eight Arab countries, besides the different population sizes between Arab countries, which indicates that our findings might be a little skewed. Third, the wide variation of healthcare systems among Arab countries in terms of unequal resource allocation, the diversity of access to healthcare services, and the differences in healthcare management strategies [79] could impact the predictors of vaccine acceptance and COVID-19 vaccine acceptance rate. In addition, each country had its own policies and protocols in obligating COVID-19 vaccine administration that we could not assess and may have influenced the opinions of the HCWs. Fourth, the included studies were conducted at different periods of time with significant variations in the availability of COVID-19 vaccines as well as in the governmental and employers’ encouragement to receive the vaccine. Lastly, the definition of vaccine acceptance varied across the included studies, where some of them used a binary approach of yes/no for participants to express their willingness to receive the vaccine, whereas others used the 5-point Likert scale. Such heterogeneity could have an impact on the reliability of our findings.

## Conclusion

A moderate acceptance rate of COVID-19 vaccines was reported among HCWs in the Arab World. Several predictors were associated with COVID-19 vaccine acceptance, such as being male, higher risk perception, positive attitude toward the influenza vaccine, and higher knowledge about COVID-19 vaccines. Considering the potential need for future booster doses of the COVID-19 vaccine or future pandemics, regulatory bodies should raise awareness regarding vaccine safety and efficacy as well as tailor their efforts to target HCWs who would consequently influence the general public with their attitude and perceptions of the vaccines. Future research could focus on exploring the reasons behind the low COVID-19 vaccine acceptance rate among the up-mentioned factors including female HCWs and addressing effective solutions and interventions to upcoming the barriers.

## Supporting information

S1 File Minimal data set. (DOCX)

S1 Checklist PRISMA 2020 checklist. (DOCX)

S1 Table Assessment of methodological quality of included studies using JBI critical appraisal checklist for analytical cross-sectional studies. (DOCX)
