# Supplementary material for: COVID-19 vaccine hesitancy among healthcare workers in Arab Countries: A systematic review and meta-analysis
Source: PLoS One. 2024 Jan 2;19(1):e0296432. doi: 10.1371/journal.pone.0296432 (PMC10760888; doi:10.1371/journal.pone.0296432)
Supplement: S1 File — (DOCX) [file pone.0296432.s001.docx]

Minimal data set

|  | **Author** | **Country** | **Number of Participants Accepting the Vaccine** | | | **Total Number of Participants (i.e., Sample Size)** | | |
| --- | --- | --- | --- | --- | --- | --- | --- | --- |
|  |  |  | **Total** | **Male** | **Female** | **Total** | **Male** | **Female** |
|  | Ahmed et al. | SA | 131 | 45 | 70 | 236 | 67 | 169 |
|  | Al Awaidy et al. | Oman | 264 | 144 | 120 | 608 | 226 | 382 |
|  | Albahri et al. | UAE | 104 | **NR** | **NR** | 176 | 18 | 158 |
|  | Aldosary et al. | SA | 236 | **NR** | **NR** | 334 | 25 | 309 |
|  | Alhofaian et al. | SA | 298 | 98 | 200 | 383 | 117 | 266 |
|  | AlKetbi et al. | UAE | 2525 | 1007 | 1518 | 2832 | 1084 | 1748 |
|  | Al-Sanafi et al. | Kuwait | 849 | 359 | 490 | 1019 | 393 | 626 |
|  | Aloweidi et al. | Jordan | 131 | **NR** | **NR** | 287 | 97 | 190 |
|  | Arif et al. | SA | 529 | **NR** | **NR** | 529 | 167 | 362 |
|  | Baghdadi et al. | SA | 222 | 107 | 115 | 363 | **NR** | **NR** |
|  | Barry et al. | SA | 1058 | 310 | 634 | 1512 | 568 | 944 |
|  | Belkebir et al. | Palestine | 15 | **NR** | **NR** | 46 | 13 | 33 |
|  | EI Kibbi et al. | Arab World | 1237 | **NR** | **NR** | 1517 | **NR** | **NR** |
|  | Elhadi et al. | Libya | 1781 | **NR** | **NR** | 2215 | 743 | 1472 |
|  | Elharake et al. | SA | 15299 | 8619 | 6680 | 23582 | 12365 | 11217 |
|  | Elkhayat et al. | Egypt | 142 | 89 | 53 | 341 | 131 | 210 |
|  | EI-Sokkary et al. | Egypt | 80 | 29 | 51 | 308 | **NR** | **NR** |
|  | Fares et al. | Egypt | 81 | 28 | 52 | 385 | 72 | 313 |
|  | Hammam et al. | Egypt | 57 | **NR** | **NR** | 187 | 28 | 159 |
|  | Hershan et al. | SA | 104 | 88 | 16 | 186 | 136 | 50 |
|  | Khalis et al. | Morocco | 119 | **NR** | **NR** | 170 | **NR** | **NR** |
|  | Khamis et al. | Khamis | 176 | **NR** | **NR** | 433 | 139 | 304 |
|  | Kumar et al. | Qatar | 619* | **NR** | **NR** | 1414 | **NR** | **NR** |
|  | Lataifeh et al.^$^ | Jordan | 233* | 96 | 137 | 364 | 135 | 229 |
|  | Luma et al. | Iraq | 1229 | **NR** | **NR** | 1704 | 978 | 726 |
|  | Maqsood et al. | SA | 996 | **NR** | **NR** | 1031 | 281 | 750 |
|  | Maraqa et al. | Palestine | 438 | 188 | 250 | 1159 | 430 | 729 |
|  | Nasr et al. | Lebanon | 455 | **NR** | **NR** | 529 | 292 | 237 |
|  | Noushad et al. | SA | 433* | 238 | 195 | 674 | 350 | 324 |
|  | Qattan et al. | SA | 340 | 228 | 112 | 673 | 405 | 268 |
|  | Qunaibi et al. | multinational | 1522* | 1049 | 473 | 5708 | 3171 | 2537 |
|  | Rabi et al. | Palestine | 517 | **NR** | **NR** | 638 | 115 | 523 |
|  | Saddik et al. | UAE | 312* | **NR** | **NR** | 517 | 187 | 330 |
|  | Sharaf et al. | Egypt | 78 | 19 | 59 | 171 | 26 | 145 |
|  | Shehata | Egypt | 495* | 223 | 272 | 1268 | 515 | 753 |
|  | Temsah et al. | SA | 352 | **NR** | **NR** | 1058 | 354 | 704 |
|  | Yassin et al. | Sudan | 254* | 102 | 152 | 400 | 156 | 244 |
|  | Youssef et al. | Lebanon | 1044 | **NR** | **NR** | 1800 | 593 | 1209 |
|  | Zammit et al. | Tunisia | 237 | 72 | 155 | 493 | 131 | 346 |

NR = Not reported

^*^ Corrected

$ not statistically significant
