# Supplementary material for: COVID-19 vaccine hesitancy among healthcare workers in Arab Countries: A systematic review and meta-analysis
Source: PLoS One. 2024 Jan 2;19(1):e0296432. doi: 10.1371/journal.pone.0296432 (PMC10760888; doi:10.1371/journal.pone.0296432)
Supplement: S1 Table — (DOCX) [file pone.0296432.s003.docx]

Table S1. Assessment of methodological quality of included studies using JBI critical appraisal checklist for analytical cross-sectional studies

| **Author/Date, Country** | **Alhofaian/2021, SA** | **Alobaidi/2022, SA** | **Arif/2021, SA** | **Baghdadi/2021, SA** | **Barry/2021, SA** | **Elharake/2021, SA** | **Hershan/2021, SA** | **Maqsood/2022, SA** | **Qattan/2021, SA** | **Temsah/2021, SA** | **Temsah/2021, SA** |
| --- | --- | --- | --- | --- | --- | --- | --- | --- | --- | --- | --- |
| **JBI criteria:** (Yes, No, Unclear, NA) |  | | | | | | | | | | |
| 1. Were the criteria for inclusion in the sample clearly defined? | Yes | Yes | Yes | Yes | Yes | Yes | Yes | Yes | Yes | Yes | Yes |
| 1. Were the study subjects and the setting described in detail? | Yes | Yes | Yes | Yes | Yes | Yes | Yes | Yes | Yes | Yes | Yes |
| 1. Was the exposure measured in a valid and reliable way? | NA | NA | NA | NA | NA | NA | NA | NA | NA | NA | NA |
| 1. Were objective, standard criteria used for measurement of the condition? | NA | NA | NA | NA | NA | NA | NA | NA | NA | NA | NA |
| 1. Were confounding factors identified? | No | Yes | Yes | Yes | Yes | Yes | Yes | Yes | Yes | Yes | Yes |
| 1. Were strategies to deal with confounding factors stated? | No | Yes | Yes | Yes | Yes | Yes | Yes | Yes | Yes | Yes | Yes |
| 1. Were the outcomes measured in a valid and reliable way? | Yes | Unclear | Yes | Yes | Yes | Unclear | Yes | Yes | Unclear | Yes | Yes |
| 1. Was appropriate statistical analysis used? | No | Yes | Yes | Yes | Yes | Yes | Yes | Yes | Yes | Yes | Yes |
| **Overall risk of bias** (Low^1^, Medium^2^, High^3^) | Moderate | Low | Low | Low | Low | Low | Low | Low | Low | Low | Low |

^1^Low risk of bias: < 3 high-risk /unclear criterion, ^2^Moderate risk of bias: 3-4 high risk/unclear criterion, ^3^High risk of bias: 5-6 high risk/unclear criterion

JBI, Joanna Briggs Institute; NA: not applicable.

Cont. Assessment of methodological quality of included studies using JBI critical appraisal checklist for analytical cross-sectional studies

| **Author/Date, Country** | **Yassssin/2022, Sudan** | **Al Awaidy/2020, Oman** | **Khamis/2022, Oman** | **Maraqa/2021, Palestine** | **Belkebir/2021, Palestine** | **Rabi/2021, Palestine** | **Kumar/2021, Qatar** | **Zammit/2022, Tunisia** | **Albahri/2020, UAE** | **AlKetbi/2021, UAE** |
| --- | --- | --- | --- | --- | --- | --- | --- | --- | --- | --- |
| **JBI criteria:** (Yes, No, Unclear, NA) |  | | | | | | | | | |
| 1. Were the criteria for inclusion in the sample clearly defined? | Yes | Yes | Yes | Yes | Yes | Yes | Yes | Yes | Yes | Yes |
| 1. Were the study subjects and the setting described in detail? | Yes | Yes | Yes | Yes | Yes | Yes | Yes | Yes | Yes | Yes |
| 1. Was the exposure measured in a valid and reliable way? | NA | NA | NA | NA | NA | NA | NA | NA | NA | NA |
| 1. Were objective, standard criteria used for measurement of the condition? | NA | NA | NA | NA | NA | NA | NA | NA | NA | NA |
| 1. Were confounding factors identified? | Yes | Yes | Yes | Yes | No | Yes | Yes | Yes | Yes | Yes |
| 1. Were strategies to deal with confounding factors stated? | Yes | Yes | Yes | Yes | No | Yes | Yes | Yes | Yes | Yes |
| 1. Were the outcomes measured in a valid and reliable way? | Yes | Yes | Unclear | Yes | Yes | Yes | Yes | Yes | Yes | Unclear |
| 1. Was appropriate statistical analysis used? | Yes | Yes | Yes | Yes |  | Yes | Yes | Yes | Yes | Yes |
| **Overall risk of bias** (Low^1^, Medium^2^, High^3^) | Low | Low | Low | Low |  | Low | Low | Low | Low | Low |

^1^Low risk of bias: < 3 high-risk /unclear criterion, ^2^Moderate risk of bias: 3-4 high risk/unclear criterion, ^3^High risk of bias: 5-6 high risk/unclear criterion

JBI, Joanna Briggs Institute; NA: not applicable.

Cont. Assessment of methodological quality of included studies using JBI critical appraisal checklist for analytical cross-sectional studies

| **Author/Date, Country** | **Aloweidi /2021, Jordan** | **Hamdan-**  **Mansour/2022,**  **Jordan** | **Lataifeh/2022,**  **Jordan** | **Qunaibi/2021, multinational** | **Al-Sanafi/2021,**  **Kuwait** | **Nasr/2021, Lebanon** | **Youssef/2022, Lebanon** | **Elhadi/2021,**  **Libya** | **Khalis/2021, Morocco** | **Ahmed/2021,**  **Saudi Arabia** | **Aldosary/2021,**  **Saudi Arabia** | **Alhasan/2021,**  **Saudi Arabia** |
| --- | --- | --- | --- | --- | --- | --- | --- | --- | --- | --- | --- | --- |
| **JBI criteria:** (Yes, No, Unclear, NA) |  | | | | | | | | | | | |
| 1. Were the criteria for inclusion in the sample clearly defined? | Yes | Yes | No | Yes | Yes | Yes | Yes | Yes | Yes | Yes | Yes | No |
| 2. Were the study subjects and the setting described in detail? | Yes | No | Yes | Yes | Yes | Yes | Yes | Yes | Yes | No | Yes | Yes |
| 3. Was the exposure measured in a valid and reliable way? | NA | NA | NA | NA | NA | NA | NA | NA | NA | NA | NA | NA |
| 4. Were objective, standard criteria used for measurement of the condition? | NA | NA | NA | NA | NA | NA | NA | NA | NA | NA | NA | NA |
| 5. Were confounding factors identified? | Yes | Yes | Yes | Yes | Yes | Yes | Yes | Yes | Yes | Yes | No | Yes |
| 6. Were strategies to deal with confounding factors stated? | Yes | Yes | Yes | Yes | Yes | Yes | Yes | Yes | Yes | Yes | No | Yes |
| 7. Were the outcomes measured in a valid and reliable way? | Yes | Yes | Yes | Yes | Yes | Yes | Yes | Yes | Yes | Yes | Yes | Unclear |
| 8. Was appropriate statistical analysis used? | Yes | Yes | Yes | Yes | Yes | Yes | Yes | Yes | Yes | Yes | No | Yes |
| **Overall risk of bias** (Low^1^, Medium^2^, High^3^) | Low | Low | Low | Low | Low | Low | Low | Low | Low | Low | Moderate | Moderate |

^1^Low risk of bias: < 3 high-risk /unclear criterion, ^2^Moderate risk of bias: 3-4 high risk/unclear criterion, ^3^High risk of bias: 5-6 high risk/unclear criterion

JBI, Joanna Briggs Institute; NA: not applicable.

Cont. Assessment of methodological quality of included studies using JBI critical appraisal checklist for analytical cross-sectional studies

| **Author/Date, Country** | **Saddik/2022, UAE** | **Hammam/2021, Egypt** | **Fares/2021, Egypt** | **Elkhayat/2021, Egypt** | **El-Sokkary/2021, Egypt** | **El Kibbi/2021, Arab World** | **Shehata/2021, Egypt** | **Luma/2022, Iraq** | **Sharaf/2022, Egypt** | **Noushad/2021, SA** | **Al-Metwali/2021, Iraq** |
| --- | --- | --- | --- | --- | --- | --- | --- | --- | --- | --- | --- |
| **JBI criteria:** (Yes, No, Unclear, NA) |  | | | | | | | | | | |
| 1. Were the criteria for inclusion in the sample clearly defined? | Yes | Yes | Yes | Yes | Yes | Yes | Yes | Yes | Yes | Yes | Yes |
| 1. Were the study subjects and the setting described in detail? | Yes | Yes | Yes | Yes | Yes | Yes | Yes | Yes | Yes | Yes | Yes |
| 1. Was the exposure measured in a valid and reliable way? | NA | NA | NA | NA | NA | NA | NA | NA | NA | NA | NA |
| 1. Were objective, standard criteria used for measurement of the condition? | NA | NA | NA | NA | NA | NA | NA | NA | NA | NA | NA |
| 1. Were confounding factors identified? | Yes | No | Yes | Yes | No | Yes | No | Yes | Yes | Yes | Yes |
| 1. Were strategies to deal with confounding factors stated? | Yes | No | Yes | Yes | No | Yes | No | Yes | Yes | Yes | Yes |
| 1. Were the outcomes measured in a valid and reliable way? | Yes | Yes | Unclear | Unclear | Yes | Yes | Yes | Unclear | Yes | Yes | Yes |
| 1. Was appropriate statistical analysis used? | Yes | No | Yes | Yes | No | Yes | No | Yes | Yes | Yes | Yes |
| **Overall risk of bias** (Low^1^, Medium^2^, High^3^) | Low | Moderate | Low | Low | Moderate | Low | Moderate | Low | Low | Low | Low |

^1^Low risk of bias: no high-risk /unclear criterion; ^2^Moderate risk of bias: one or two high risk/unclear criterion; ^3^High risk of bias: more than two high risk/unclear criterion.

JBI, Joanna Briggs Institute; NC, not clear.
